# A Sequential Model of Host Cell Killing and Phagocytosis by *Entamoeba histolytica*

**DOI:** 10.1155/2011/926706

**Published:** 2011-01-20

**Authors:** Adam Sateriale, Christopher D. Huston

**Affiliations:** ^1^Department of Medicine, College of Medicine, The University of Vermont, Room 320 Stafford Hall, 95 Carrigan Drive, Burlington, VT 05405, USA; ^2^Cell and Molecular Biology Graduate Program, College of Medicine, The University of Vermont, Burlington, VT 05405, USA; ^3^Department of Microbiology and Molecular Genetics, College of Medicine, The University of Vermont, Room 320 Stafford Hall, 95 Carrigan Drive, Burlington, VT 05405, USA

## Abstract

The protozoan parasite *Entamoeba histolytica* is responsible for invasive intestinal and extraintestinal amebiasis. The virulence of *Entamoeba histolytica* is strongly correlated with the parasite's capacity to effectively kill and phagocytose host cells. The process by which host cells are killed and phagocytosed follows a sequential model of adherence, cell killing, initiation of phagocytosis, and engulfment. This paper presents recent advances in the cytolytic and phagocytic processes of *Entamoeba histolytica* in context of the sequential model.

## 1. Introduction

*Entamoeba histolytica* is an enteric parasite that colonizes the human intestinal lumen and has the capacity to invade the epithelium. Although 90% of amebic infections are asymptomatic and self-limiting, there are an estimated 50 million cases of invasive infection annually [1, 2]. According to the WHO, *Entamoeba histolytica* is ranked third as a cause of death among parasites with 100,000 estimated deaths annually [1]. The morbidity and mortality of this parasite is primarily seen in developing countries. Ingestion of contaminated food or water containing infectious cysts leads to excystation in the intestine. Each cyst produces eight motile trophozoites, which colonize the host's colon. In those cases where the infection is not self limiting, amebic dysentery and liver abscess formation can occur [2].

The process of invasion and hepatic abscess formation has no apparent advantage for *Entamoeba histolytica* [3]. The logical question would then be why did this organism evolve to be a pathogen and not a commensal like its noninvasive cousin, *Entamoeba dispar*? One theory of *Entamoeba histolytica's* origin of virulence is coincidental evolution. Host cells may have recognition patterns similar to those of enteric bacteria that the parasite has evolved to identify. *Entamoeba histolytica* has been shown to preferentially phagocytose cells coated with collectins, C-type lectins involved in recognition of ligands that are common to both bacteria and apoptotic cells [4]. An effective hijacking of the host's own innate immune system to increase phagocytosis may have led to an invasive phenotype. In further support of this theory, Ghosh and Samuelson [3] have shown that several signaling proteins required for *Entamoeba histolytica's* virulence are also utilized to kill and phagocytose bacteria. Another seemingly plausible explanation is that *Entamoeba histolytica*'s invasive phenotype arose in response to host defense mechanisms [5]. Directed apoptosis and subsequent phagocytosis may serve to limit host inflammatory mechanisms by suppressing necrosis and subsequent Th1-type immunity [6]. Cysteine proteases that are known to degrade host extracellular matrix also protect *Entamoeba histolytica* from complement, secretory IgA, and serum IgG [7–9].

**Figure 1:**
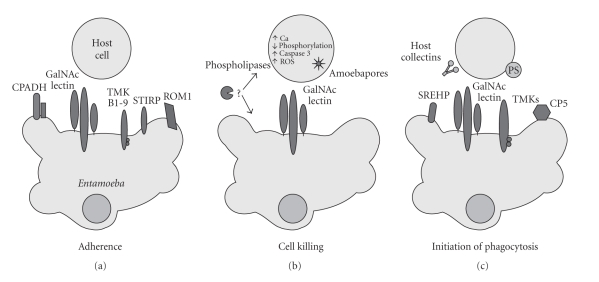
Sequential model of cell killing and phagocytosis by *Entamoeba histolytica*. Adherence, cell killing, and initiation of phagocytosis leading to engulfment of host cells are depicted from left to right. Abbreviations: cysteine protease adhesin (CPADH), transmembrane kinase (TMK), serine-threonine-isoleucine rich protein (STIRP), reactive oxygen species (ROS), serine-rich *Entamoeba histolytica *protein (SREHP), cysteine protease 5 (CP5), and phosphatidylserine (PS).

While the evolutionary basis behind virulence is uncertain, the mechanism behind virulence is slowly becoming clearer. Invasion by *Entamoeba histolytica* is strongly correlated with the parasite's capacity to kill and phagocytose host cells [10–13]. The function of this review is to highlight some of the recent advances in understanding the mechanism of cell killing and phagocytosis, and to place these findings in the context of previous knowledge. For the purpose of this review, cell killing and phagocytosis have been organized in a sequential model involving (i) adherence to the host cell surface, (ii) contact-dependent cell killing, (iii) initiation of phagocytosis, and (iv) engulfment (see Figure 1).

## 2. Adherence

The d-galactose/N-acetyl-d-galactosamine- (GalNAc-) specific lectin is the major amebic surface adhesin responsible for adherence to intestinal mucus and host cells [14]. The GalNAc lectin is composed of a light subunit (Lgl), heavy subunit (Hgl), and a noncovalently bound intermediate subunit (Igl) [15, 16]. The light and heavy subunits are linked via a disulfide bond and exist predominantly at the parasite cell membrane as a 260 kDa heterodimer [15]. The heavy subunit contains a carbohydrate recognition domain (CRD) that recognizes d-galactose and N-acetyl-d-galactosamine [17]. MUC-2, the predominant mucin in the host intestine, is bound by the GalNAc lectin with high affinity (*K*_*d*_ = 8.2 × 10^−11^ M), allowing for *Entamoeba histolytica* to colonize mucosal surfaces [18, 19]. The CRD also recognizes host cell surface protein glycoconjugates and inhibition of adherence to host cells has been shown using monoclonal antibodies that bind the CRD specifically [20, 21]. Host cell adherence can also be strongly inhibited using *μ*M concentrations of either galactose or N-acetyl-d-galactosamine [14, 22, 23]. Inhibition of adherence through the GalNAc lectin invariably leads to a subsequent decrease in host cell cytotoxicity [23]. Tetracycline-regulated expression of a truncated intracellular domain of the GalNAc lectin heavy subunit has been shown to significantly decrease adherence to host cells *in vitro * [24]. These data suggest that the lectin participates in outside-to-inside signaling, which is likely through the *β*2 integrin homologous intracellular domain of the GalNAc heavy subunit. These functions in adhesion and signaling place the GalNAc lectin firmly at the nexus of virulence, though there are other *Entamoeba histolytica* proteins that have been implicated in adherence.

The EhCPADH complex is a 124 kDa heterodimer formed by a cysteine protease (EhCP112) and an adhesin (EhADH112). Targeted monoclonal antibodies to the C-terminus adhesion epitope of ADH112 results in greater than 50% reduced adherence to host cells, and ensuing decreases in cytotoxicity and phagocytosis [25]. ADH112 has three putative transmembrane domains, a putative Bro1 domain, and an intracellular domain with potential phosphorylation sites [26]. It will be interesting to see whether targeted mutations to the intracellular region or a truncated version of this protein produce a parasite with diminished adherence. The ADH112 intracellular domain is highly divergent from that of the GalNAc lectin heavy subunit [26]. Adhesion signaling mechanisms of these complexes are, therefore, likely to be distinct. 

Many of the proteins recently implicated in adherence have arisen from genomic and transcriptomic analyses of *Entamoeba histolytica* and nonvirulent *Entamoeba*. Sequencing of the *Entamoeba histolytica* genome has led to many new discoveries, truly advancing the field of *Entamoeba* research in a manner not seen since Diamond et al. first axenically cultured the parasite [27–29]. One such discovery is STIRP (serine-threonine-isoleucine rich protein), a protein family exclusively expressed in virulent strains of *Entamoeba, in vitro*. shRNA-mediated silencing of the STIRP family led to a 35% decrease in adhesion to host cells and a subsequent reduction in cytotoxicity [30]. ROM1 is a serine protease functionally related to the rhomboid proteases first identified in *Drosophila melanogaster* [31, 32]. Rhomboid proteases are seven-pass transmembrane proteases with the ability to cleave transmembrane proteins at their transmembrane domain [33]. The ROM1 gene appears to be the only rhomboid protease expressed by both *Entamoeba histolytica *and* Entamoeba dispar*. shRNA-mediated silencing of ROM1 reduced adhesion to healthy Chinese hamster ovary (CHO) cells, but not to apoptotic CHO cells, the mechanism of which is still to be determined. It is hypothesized that the ROM1 protease could be involved in cleavage and activation of amebic transmembrane proteins involved in adherence and phagocytosis. ROM1 silenced ameba were shown to have an ordinary amount of GalNAc lectin at their cell surface, but other amebic adhesins may be modulated by ROM1 [31]. There is experimental evidence of at least one additional *Entamoeba histolytica* surface lectin activity involved in phagocytosis [34].

Another recently described potential adhesin is TMKB1-9, a member of a large family of transmembrane kinases (the relevance of which is more thoroughly discussed later) [35]. The expression of TMKB1-9 was shown, quite conclusively, to correlate with decreased adherence to and destruction of CHO cell monolayers. Intriguingly, the expression of TMKB1-9 also correlated to serum content in the culture medium, suggesting a possible mechanism for sensing environmental conditions [36]. As this exciting new research unfolds, we shall hopefully better understand what serum component(s) is regulating TMKB1-9 expression, and how TMKB1-9 modulates cell adherence.

Trophozoites of *Entamoeba histolytica* express GPI-anchored lipoglycoconjugates on their cell surface, referred to as lipopeptidophosphoglycans or EhLPPG [37, 38]. These molecules have been implicated in host-parasite interactions based on the finding that nonvirulent and virulent strains of *Entamoeba histolytica* express different amounts and structures of EhLPPG [39–42]. Recent research has shown that EhLPPG are the primary NKT cell ligands, helping to explain why CD1d−/− mice show significantly larger liver abscesses [43, 44]. Marinets et al. [45] found that passive immunization with antibody to LPPG conferred protection from invasive amebiasis in the severe combined immunodeficient (SCID) mouse model of hepatic abscess. This effect was also seen using a SCID intestinal xenograph model of invasion [46]. LPPG antibody also caused agglutination of ameba *in vitro*, which may have been a confounding factor in an earlier report showing an LPPG antibody-mediated decrease in adherence [47]. LPPG may be vitally important in immune recognition, but the role it plays in host cell-parasite adherence remains uncertain. Finally, the lysine and glutamic acid-rich protein, KERP1, remains an attractive potential adhesion, as it has been shown to bind epithelial cells and is absent in the *Entamoeba dispar* genome [48]. Its role in adhesion has yet to be formally tested, but KERP1 has recently been evidenced to play a role in liver abscess formation [49].

## 3. Cell Killing

The GalNAc lectin is a striking example of a crossover function between adherence and cell killing. Antibodies targeting the heavy subunit (Hgl) on a separate domain from the CRD decrease cell killing by approximately 50% [50]. It should be noted that exclusion of any adherence protein from the subsequent processes of cell killing and initiation of phagocytosis does not rule out their involvement, only a lack of evidence to suggest significant involvement in the latter two. It is quite possible that many of the proteins involved in the recognition of healthy host cells are also involved in the cytolysis and/or recognition of apoptotic cells, much like the GalNAc lectin.

The *Entamoeba histolytica* genome encodes three amoebapore proteins that can be secreted upon contact, and the purified proteins cause target host cell membrane permeability at *μ*M concentrations [51, 52]. When inserted into host cell membrane, amoebapore proteins oligomerize through peptide-peptide interactions to produce ion channels [53]. Antisense silencing of amoebapore A expression significantly impairs *Entamoeba histolytica's* ability to kill baby hamster kidney (BHK) cells, assayed by trypan blue exclusion [54]. The G3 strain of *Entamoeba histolytica* has an almost complete transcriptional silencing of the amoebapore A protein [55]. The G3 strain was also shown to be deficient in cell monolayer destruction and incapable of forming liver abscess in the hamster model of hepatic abscess [55]. Conversely, the G3 strain produced abscesses, though of smaller size, in the SCID mouse model [56]. The authors speculate this difference may have been due to the increased susceptibility of the SCID mice, variable timing of liver assessment, or variation in the role that amoebapore plays in different animal models. 

While target host cells and bacteria are susceptible to amoebapore, *Entamoeba histolytica* is surprisingly resistant at *μ*M concentrations. Experiments using liposomes with *Entamoeba histolytica* cell membrane composition demonstrated that the phospholipid composition of the parasite plasma membrane, along with its high cholesterol content, prevents binding of fluorescently labeled amoebapore [57]. The plasma membrane of *Entamoeba histolytica* is also resistant to another protein implicated in host cell killing, phospholipase [58]. Pharmacological inhibitors of eukaryotic phospholipase A significantly reduced CHO cell killing, as measured by trypan blue exclusion criteria [58]. The predominant phospholipid found on the *Entamoeba* cell membrane is ceramide aminoethylphosphonate (CAEP), which is a phospholipase resistant species of phospholipid [59, 60]. While phosphonolipids have been found in small amounts in various mammals, such large amounts of CAEP have only been seen in marine bacteria, gastropods, and bivalve mollusks [61]. CAEP was also detected in the plasma membrane of *Entamoeba histolytica*'s reptilian relative, *Entamoeba invadens *[62]. It is possible that CAEP confers resistance to* Entamoeba histolytica*'s resident phospholipases. 

Following contact with *Entamoeba histolytica* host cells undergo the morphological and phenotypic changes of apoptosis, including nuclear chromatin condensation, DNA fragmentation, and membrane blebbing [63]. These cells stain positive by terminal deoxynucleotidyl-transferase-mediated dUTP-biotin nick-end labeling (TUNEL) and by annexin V, indicating DNA degradation and phosphatidylserine increases on the outer leaflet of the host cell plasma membrane [64]. Although one study has shown necrotic features of *Entamoeba histolytica*-induced cell death, predominant amount of the literature supports an apoptotic result [65–71]. The mechanism by which this host-cell apoptosis is initiated in a variety of different cell types is still unclear, but there are some common factors. Target cells show a sustained increase in intracellular Ca^2+^ concentration, protein tyrosine dephosphorylation, and caspase 3 activation following contact with *Entamoeba histolytica * [66, 72, 73]. Recent work has shown that pretreatment of Jurkat lymphocytes with the calpain inhibitor calpeptin leads to a decrease in protein tyrosine dephosphorylation. It is hypothesized that the increase in host cell intracellular Ca^2+^ concentration activates calpain, which cleaves and activates host SHP-1 and SHP-2. SHP-1 and SHP-2 then act as protein tyrosine phosphatases. Although calpeptin pretreatment leads to a decrease in protein tyrosine dephosphorylation, it is insufficient to halt ensuing apoptosis [74]. Caspase 8 deficiency and caspase 9 inhibition have likewise been shown to be ineffective in abrogating apoptosis in target Jurkat lymphocytes. Conversely, the caspase 3 inhibitor Ac-DEVD-CHO was found to block Jurkat cell apoptosis, measured by DNA fragmentation and 51Cr release [66]. In a C57BL/6 mouse model of hepatic abscess, *Entamoeba histolytica*-induced apoptosis was also found to be Fas/Fas ligand independent [64]. These findings support a Fas/Fas ligand and caspase 8/9 independent activation of caspase 3. 

Recent research using a CBA mouse model of colitis has shown that intraperitoneal injection with the pan-caspase inhibitor ZVAD reduced the mouse parasite burden and, further, that caspase 3 knockout C57BL/6 mice showed an even lower parasite burden [6]. The fact that caspase 3 knockout mice were not fully protected from *Entamoeba* invasion suggests a possible second mechanism of cell death. Sim et al. [70] have shown in neutrophils that intracellular reactive oxygen species (ROS) are induced upon contact from *Entamoeba histolytica*. This induction also coincides with an increasing ERK1/2 activation. Incubation with a MEK1 inhibitor decreased ERK1/2 activation and neutrophil apoptosis. Recent work from this group indicates that apoptosis in neutrophils is also inhibited by host cell preincubation with monoclonal antibodies to CD18 [75]. CD18 is a *β*2 integrin that mediates neutrophil adhesion and is known to promote activation of NADPH oxidase [76]. Treatment with an NADPH oxidase inhibitor also partially decreased neutrophil apoptosis, as measured by annexin-V staining of phosphatidylserine [70]. Previous studies have shown GalNAc lectin deposition on target host cell membranes following parasite contact [77]. It is interesting to speculate that, if integrated into the host cell membrane, the *β*2 integrin domain of the GalNAc lectin heavy subunit may be capable of stimulating NADPH oxidase. Whether the ROS-dependent pathway and the caspase 3-dependent pathway are part of the same mechanism of apoptosis or separate, the end result is membrane blebbing and increased phosphatidylserine exposure on the outer leaflet of the host plasma membrane [13, 67].

## 4. Initiation of Phagocytosis

Experiments have shown, conclusively, that *Entamoeba histolytica* more readily phagocytoses host cells that have already undergone apoptosis [13, 67]. Apoptotic Jurkat lymphocytes and Ca^2+^ ionophore-treated erythrocytes are both phagocytosed at a higher rate than their viable counterparts. Jurkat lymphocytes made artificially apoptotic by insertion of phosphatidylserine into the outer leaflet are also phagocytosed by *Entamoeba histolytica* at a higher rate [67]. When healthy Jurkat lymphocytes were incubated with *Entamoeba histolytica in vitro*, caspase 3 activity was detected by immunofluorescence using an antiactive caspase 3 antibody in virtually all intact cells ingested [67]. Thus, apoptosis appears to be a requirement for phagocytosis to occur, though it remains possible that viable cells are just engulfed less efficiently. 

Galactose inhibition of the GalNAc lectin leads to a 22% reduction in amebic adherence to Ca^2+^ ionophore-treated erythrocytes, in contrast to healthy erythrocytes which show approximately 81% reduction in adherence [13]. Similarly, d-galactose inhibits adherence to apoptotic Jurkat lymphocytes inefficiently [67]. These results clearly implicate other *Entamoeba histolytica* receptors in adhesion to apoptotic host cells and initiation of phagocytosis. 

Ideal candidates for apoptotic receptors are members of the *Entamoeba histolytica* transmembrane kinase family of proteins. *Entamoeba histolytica* has over 90 transmembrane kinases (TMKs), categorized into subfamilies (A, B1-3, C, D1-2, E, F) based on signature motifs in their kinase domains [35]. Single-cell microarray analysis of *Entamoeba histolytica* has shown that multiple TMKs are expressed by individual parasites* in vitro *[78]. A small subset of these proteins has been characterized, thus far, with surprising results. Certain members of the TMK family have been implicated in proliferation, possibly due to signaling involving the extracellular milieu [36, 78, 79]. TMKB1-9 levels have been shown to correlate with serum levels in culture media; in fact, many of the TMKs have expression patterns that fluctuate over time [35, 36]. Other TMKs have exhibited a role in the uptake of host cells, specifically in the recognition of apoptotic host cells [78, 80]. Expression of a carboxy-truncated version of TMK39, possessing only extracellular and transmembrane domains, decreased uptake of apoptotic Jurkat lymphocytes by approximately 50% [78]. Similarly, expression of a truncated version of TMKB3-96 (PATMK) decreased uptake of Ca^2+^ ionophore-treated erythrocytes [80]. This decrease was also shown using shRNA-mediated knockdown and using polyclonal antiserum specific for PATMK, which localized to the phagocytic cup during erythrophagocytosis. 

Exactly what these TMKs are recognizing on apoptotic cells is unknown. Phosphatidylserine exposure is a hallmark of host cell apoptosis, making it a strong candidate ligand [81, 82]. Annexin V masking of phosphatidylserine on apoptotic erythrocytes leads to a decrease in phagocytosis [13]. Annexin V treatment along with galactose inhibition of the GalNAc lectin also leads to an astonishing >95% reduction in erythrophagocytosis. If phosphatidylserine were the only driving force behind apoptotic cell recognition, then annexin V treatment of other apoptotic cell types should also decrease phagocytosis. Interestingly, this effect is not seen. Annexin V treatment of Jurkat lymphocytes does not affect the rate of phagocytosis *in vitro* (C. Huston, unpublished data). These findings lead us to believe that, while phosphatidylserine may be a strong signal for initiation of phagocytosis, other ligands present on nucleated apoptotic host cells must be also capable of stimulating *Entamoeba histolytica* phagocytosis. 

**Figure 2:**
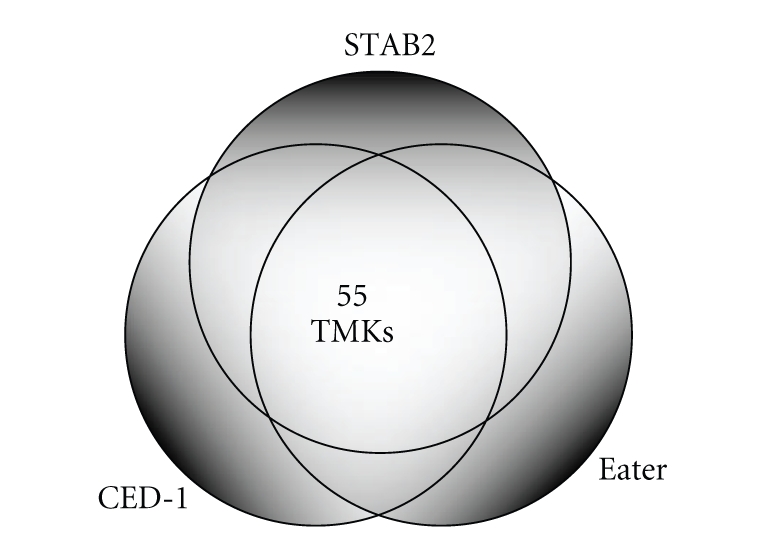
Venn diagram summarizing results of *Entamoeba histolytica* BLAST searches using the extracellular domains of CED-1 (*C. elegans*), eater (*D. melanogaster*), and STAB2 (*H. sapiens*). Fifty-five members of the *E. histolytica *transmembrane kinase gene family share significant homology to these representative scavenger receptors.

Research on macrophage uptake of apoptotic cells has shown that recognition of phosphatidylserine alone involves multiple receptors [83, 84]. As previous studies have noted, the extracellular domain of TMKs contain many epidermal growth factor- (EGF-) like repeats characteristic of scavenger receptors conserved in eukaryotes [78, 85]. A Boolean exploration of BLAST searches involving the extracellular domains of the representative scavenger receptors CED-1 (*C. elegans*), eater (*D. melanogaster*), and STAB2 (*H. sapiens*) returns 55 members of the *Entamoeba histolytica* TMK family (Figure 2). This number is remarkable considering that many of the transmembrane kinase genes encode truncated forms, lacking substantial extracellular domains [35, 79]. Proteomic analysis of the *Entamoeba histolytica* phagosome using carboxylated paramagnetic beads as bait identified 22 TMKs over various time points (Table 1) [80]. It is an attractive hypothesis that TMKs are acting as scavenger receptors, yet more research is needed to characterize TMK ligands and the downstream signaling induced. Buss et al. [78] observed heterodimerization of wild type and truncated TMKs in transfected parasites. It will be interesting to see whether TMK homodimerization alone is sufficient to initiate phagocytosis, and whether TMKs are able to dimerize with other family members.

Another large family of genes in *Entamoeba histolytica* is the cysteine proteases, of which there are 50 known members [87]. EhCP1, EhCP2, and EhCP5 appear to make up nearly 90% of the cysteine protease transcripts in cultured parasites [88, 89]. At different time points of infection, the expression of cysteine proteases can shift greatly, leading to the increase of EhCP4 and others [90]. In cultured parasites, antisense knockdown of EhCP5 resulted in a 90% decrease in cysteine protease activity compared to wild type [91]. Strangely, this strain of *Entamoeba histolytica* had a decrease in phagocytosis, while having no apparent defect in hemolytic activity or monolayer destruction. This is in stark contrast to the known roles of cysteine proteases that include degradation of extracellular matrix, mucin, complement proteins, immunoglobulins, and cytokines [7–9, 92]. EhCP5-attenuated parasites were also unable to penetrate the colonic lamina propria in an *ex vivo* human colonic model of invasion [93]. Targeted inhibitors to EhCP1 and EhCP4 have also been shown to be protective in the SCID mouse-human intestinal xenograph model and in the SCID mouse hepatic abscess model, respectively [94, 95]. The connection between cysteine proteases and phagocytosis has not been determined, but their importance for host invasion has been proven *ex vivo* and *in vivo*. The availability of pharmacologic inhibitors for cysteine proteases makes them attractive targets for drug design, and the inhibitors are potential tools to dissect the roles of individual cysteine proteases in phagocytosis. 

The serine rich *Entamoeba histolytica* protein (SREHP) was first identified based on its strong immunogenic properties, and characterized as a potential parasite chemoattractant [96]. These results are perplexing considering that the SREHP does not appear to be secreted, but does show localization to the plasma membrane of *Entamoeba histolytica*. An* in vitro* screen of 43 monoclonal antibodies raised against *Entamoeba histolytica* membrane preparations identified a single antibody that inhibited phagocytosis, which was found to be specific for SREHP [97]. This antibody blocked uptake of apoptotic Jurkat lymphocytes by over 90%, and the reduction was shown to be GalNAc lectin-independent via saturating amounts of galactose. Adherence and induction of apoptosis were also reduced to a much lesser degree. The SREHP has a putative transmembrane domain but no appreciable cytoplasmic domain, implicating a possible coreceptor that is still to be identified.

The host collectins C1q, SP-A, and MBL have all been shown to be ligands that stimulate *Entamoeba histolytica *phagocytosis [4]. Structurally, the collectin family all have a collagenous N-terminal tail and a globular C-terminal head generally involved in opsonization [98]. Collectins are found throughout the host mucosal lining, including those of the intestine [99–101]. Collectin-mediated opsonization of bacteria and apoptotic host cells is stimulatory for *Entamoeba histolytica* as well as macrophages [4, 102] (A. Sateriale, unpublished data). Pretreatment with C1q increased amebic uptake of apoptotic Jurkat lymphocytes *in vitro*, but not of viable Jurkats, even though C1q was detectable on the surface of both. The localization of C1q to apoptotic Jurkat membrane blebs in these experiments indicates possible concentration dependence. C1q and MBL were also found to be chemoattractants for *Entamoeba histolytica,* via a transwell migration assay [4]. As the host collectins have been shown to be structurally similar, a single receptor may show cross-reactivity. However, a putative *Entamoeba histolytica* collectin receptor has yet to be identified.

## 5. Engulfment

The process of host-cell engulfment following initiation of phagocytosis has been shown to be actin and myosin dependent [103]. Rhodamine-labeled phalloidin localizes to the phagocytic cup during target cell ingestion, and cytochalasin D blocking of actin polymerization has been shown to inhibit phagocytosis [104–106]. An *Entamoeba histolytica* strain with a threefold overexpression of myosin 1B exhibited marked deficiency in erythrophagocytosis [107]. Recent research has also posited that *Entamoeba* lipid rafts are involved in the organization of host-cell adhesion and endocytosis [108]. In a cholesterol-rich organism such as *Entamoeba histolytica,* it is not difficult to imagine the large role lipid rafts could play in organizing pathogenic events [59]. *Entamoeba histolytica* signaling proteins that have been shown to regulate host-cell engulfment include p21 activated kinase (PAK), protein kinase C (PKC), RacA, and phosphatidylinositol 3-kinase (PI3 kinase) [3, 109, 110]. Recent proteomic research involving purified phagosomes has given supporting evidence to these observations and offers a more complete picture of the various proteins involved in amebic endocytosis [80, 111–113]. Okada and Nozaki [114] and Marion and Guillén [85] offer very concise and comprehensive reviews of the endocytosis mechanism.

## 6. Future Directions

Some of the original mysteries surrounding *Entamoeba histolytica* pathogenicity still plague researchers today. The Zulu word for *Entamoeba histolytica*-derived liver abscess is *isigwebedhla*, which translates to disease of the strong young men [115]. The cause behind the gender bias still remains unknown. This is not particularly surprising, considering that the mechanism by which *Entamoeba histolytica* causes host cell apoptosis is largely uncertain. Models for assaying parasite invasion such as the SCID mouse-human xenograph model and the recent *ex vivo* human intestinal model may allow for a better understanding of host-parasite interactions [93, 116]. While animal models are invaluable, discrepancies between species and even between strains highlight the variability of the host-parasite interface. Models better representing the parasite's natural human host may allow for a better understanding of the invasive phenotype. Many of the proteins described in this sequential model of invasion also happen to be the most immunogenic [117]. The characterization of novel proteins involved in adherence, cell killing, and phagocytosis still holds the promise of identifying future vaccine candidates.

## Figures and Tables

**Table 1:** Members of the* Entamoeba histolytica* transmembrane kinase family found in phagosome preparations at various time points [80, 86].

TMK	Pathema ID
EhTMKA-4	EHI_068720
EhTMKA-85	EHI_128430
EhTMKB1-1	EHI_103240
EhTMKB1-5	EHI_062090
EhTMKB2-14	EHI_068160
EhTMKB2-31	EHI_180320
EhTMKB2-36	EHI_074740
EhTMKB2-41	EHI_064490
EhTMKB2-75	EHI_092260
EhTMKB3-29	EHI_050820
EhTMKB3-96	EHI_167650
EhTMKC-13	EHI_025280
EhTMKC-71	EHI_030420
EhTMKD1-3	EHI_201270
EhTMKD1-40	EHI_064500
EhTMKD1-70	EHI_189290
EhTMKD1-79	EHI_180150
EhTMKD2-19	EHI_081790
EhTMKD2-44	EHI_127000
EhTMKD2-64	EHI_086050
EhTMKE-22	EHI_186990
EhTMKE-54	EHI_188110
